# A Case of a Patient with Adhesive Small Bowel Obstruction in Pregnancy after Extensive Myomectomy for Diffuse Uterine Leiomyomatosis

**DOI:** 10.1155/2022/3601945

**Published:** 2022-09-26

**Authors:** Yuri Suminaga, Mana Taki, Haruko Okamoto, Yosuke Kawamura, Yusuke Sagae, Masumi Sunada, Yoshitsugu Chigusa, Akihito Horie, Masaki Mandai, Haruta Mogami

**Affiliations:** Department of Gynecology and Obstetrics, Kyoto University, Kyoto, Japan

## Abstract

**Background:**

Diffuse uterine leiomyomatosis is a rare disease in which countless, poorly defined, and small nodules are present in most parts of the uterine myometrium. It frequently occurs in fertile women and causes infertility. A deep, median, longitudinal incision of the uterine corpus with the opening of the endometrial cavity, “extensive myomectomy,” is required to restore fertility. However, myomectomy may also be a risk factor for perinatal complications. We present a rare case of adhesive small bowel obstruction after extensive myomectomy for diffuse uterine leiomyomatosis.

**Case:**

A 37-year-old primigravida presented with sharp epigastric pain and vomiting at 21-week gestation. The patient had a history of extensive myomectomy for diffuse uterine leiomyomatosis. Abdominal radiography revealed moderate air fluid levels in the small intestine, and the patient was diagnosed with adhesive small bowel obstruction. The patient was also diagnosed with placenta previa. Bowel rest with intestinal tube was continued until delivery. Cesarean section was performed at 32-week gestation due to (i) prolonged fasting and total parenteral nutrition for conservative treatment and (ii) fear of sudden massive bleeding from placenta previa. Because the ileum was strongly adherent to the uterine scar from the previous myomectomy, adhesiolysis and enterectomy were performed. The placenta was uncomplicatedly delivered and the hemorrhage was well-controlled.

**Conclusions:**

Pregnancy with a history with extensive myomectomy for diffuse uterine leiomyomatosis should be carefully monitored because of the occasional occurrence of adhesive small intestine obstruction during pregnancy.

## 1. Introduction

Diffuse uterine leiomyomatosis is a rare and benign disease that results in enlargement of the uterus with almost complete replacement of the uterine myometrium by innumerable, poorly defined, and small nodules ranging from microscopic to 3 cm in size [1–3]. It frequently occurs in fertile women and causes infertility [4, 5]. Typical myomectomy is insufficient for diffuse uterine leiomyomatosis; a deep, median, and longitudinal incision of the uterine corpus with the opening of the endometrial cavity is required, and “extensive myomectomy” is performed to restore fertility [4, 6]. However, myomectomy could also increase the risk of other perinatal complications including postoperative adhesions.

Small bowel obstruction in pregnancy is a rare perinatal complication with an incidence of 0.001–0.003% and is a life-threatening situation for mothers and fetuses [7–10]. Small bowel obstruction is primarily caused by adhesions, hernias, inflammatory diseases, and malignancies. Adhesion is the leading cause of small bowel obstruction, even during pregnancy. A history of abdominal surgery is the most common cause of adhesions, leading to adhesive small bowel obstruction during pregnancy. Small bowel obstruction is defined as a partial or complete blockade of the small intestine [11]. The symptoms are mainly abdominal pain, vomiting, constipation, and distension, and worsen during pregnancy. Most patients are conservatively treated with nasogastric or intestinal tubes and total parenteral nutrition (TPN), whereas surgical intervention during pregnancy is sometimes required if the small intestine is strangulated. Increased maternal mortality and fetal loss have been reported in the literature [8].

In this report, we present a rare case of adhesive small bowel obstruction a year after extensive laparotomic myomectomy for diffuse uterine leiomyomatosis.

## 2. Case Report

**Figure 1:**
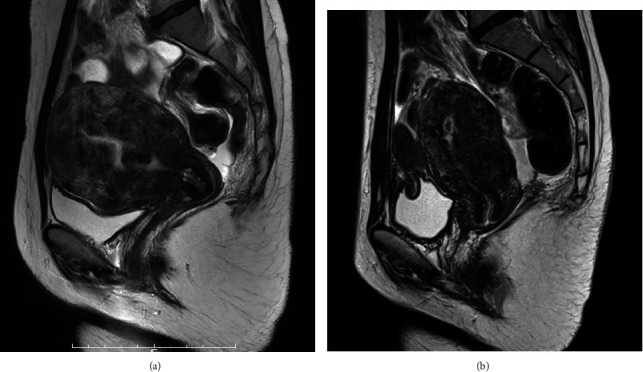
MRI T2-weighted findings of diffuse uterine leiomyomatosis (a) before and (b) after extensive myomectomy.

A 37-year-old primiparous woman presented at 21-week gestation with sharp epigastric pain and vomiting. She underwent extensive laparotomic myomectomy with a longitudinal incision of the anterior and posterior wall of the uterus for diffuse uterine leiomyomatosis and removal of 49 nodules 9 months before pregnancy that resulted from artificial insemination (Figure 1). She was afebrile and had normal blood pressure and pulse rate. She had epigastric tenderness, and her uterus was soft with no abnormal signs. Abdominal radiography revealed mild air and fluid levels in the small intestine. Sonography showed total placenta previa. She was admitted to our hospital with adhesive small intestinal obstruction and total placenta previa, which could be a postoperative complication of her previous myomectomy. One week of intravenous fluids and bowel rest improved her symptoms, and she was discharged.

**Figure 2:**
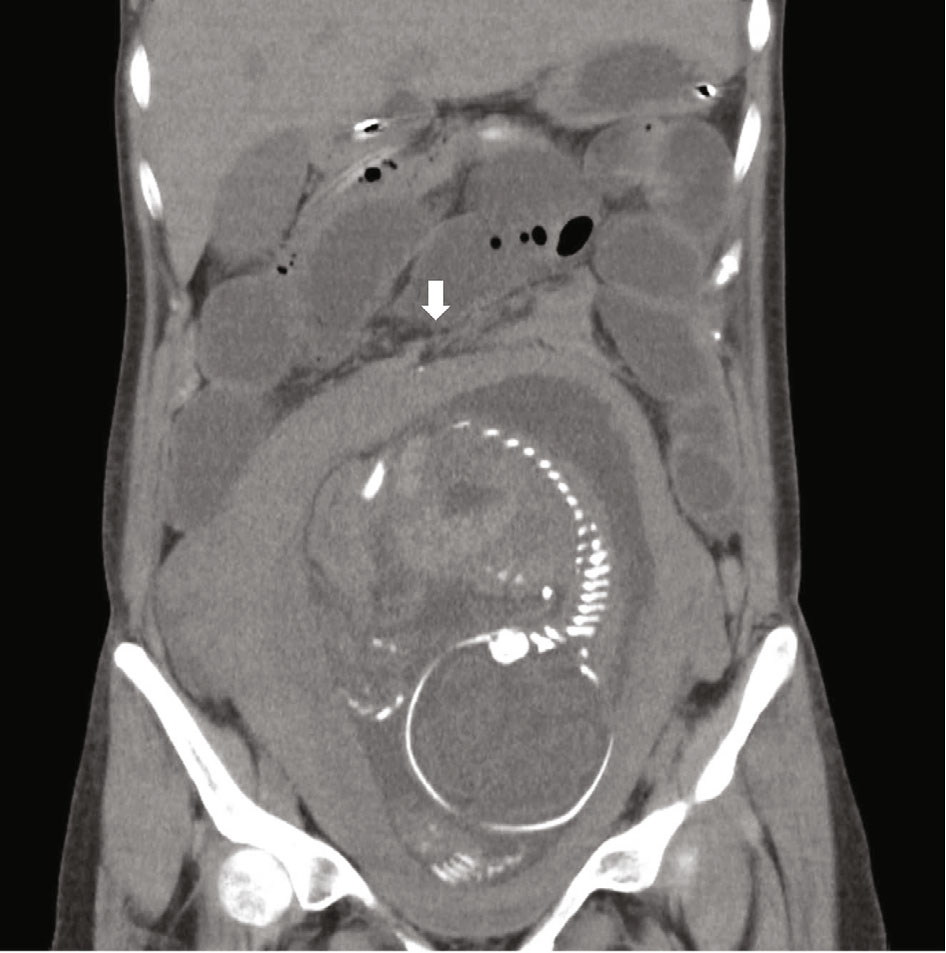
CT findings of adhesive small intestine at 26-week gestation. An arrow indicates adhesion site.

She was re-admitted again at 26-week gestation for abnormal genital bleeding due to placenta previa. The patient had no abdominal pain and normal appetite. Three days later, the patient experienced sharp epigastric pain, vomiting, distention, and constipation. Abdominal radiography showed moderate air fluid levels in the small intestine, and she was diagnosed with worsening adhesive small bowel obstruction. Bowel rest and intravenous fluids were started at the same time that tocolysis was needed because of the increasing uterine contraction caused by vomiting. Over the next 72 h, she developed fever with worsening symptoms, in which small intestine obstruction promoted bacterial translocation. Computed tomography (CT) revealed consecutive dilatation of the proximal small intestine and collapsed distal ileum adjacent to the fundus of the uterus without free air (Figure 2). The patient was managed using an intestinal tube and antibiotics. However, her abdominal pain did not resolve, and accordingly, her uterine contraction, genital bleeding, and fever increased. We discussed this with gastroenterologists and thought that it would be better to continue the conservative therapy, without surgical treatment. We inserted the intestinal tube deeper into the adhesion site (total 245 cm in length) at 28-week gestation, and her symptoms, including fever and uterine contraction, gradually resolved. Unexplained oligohydramnios occurred 4 days after fever, but gradually resolved within 10 days as her symptoms improved.

**Figure 3:**
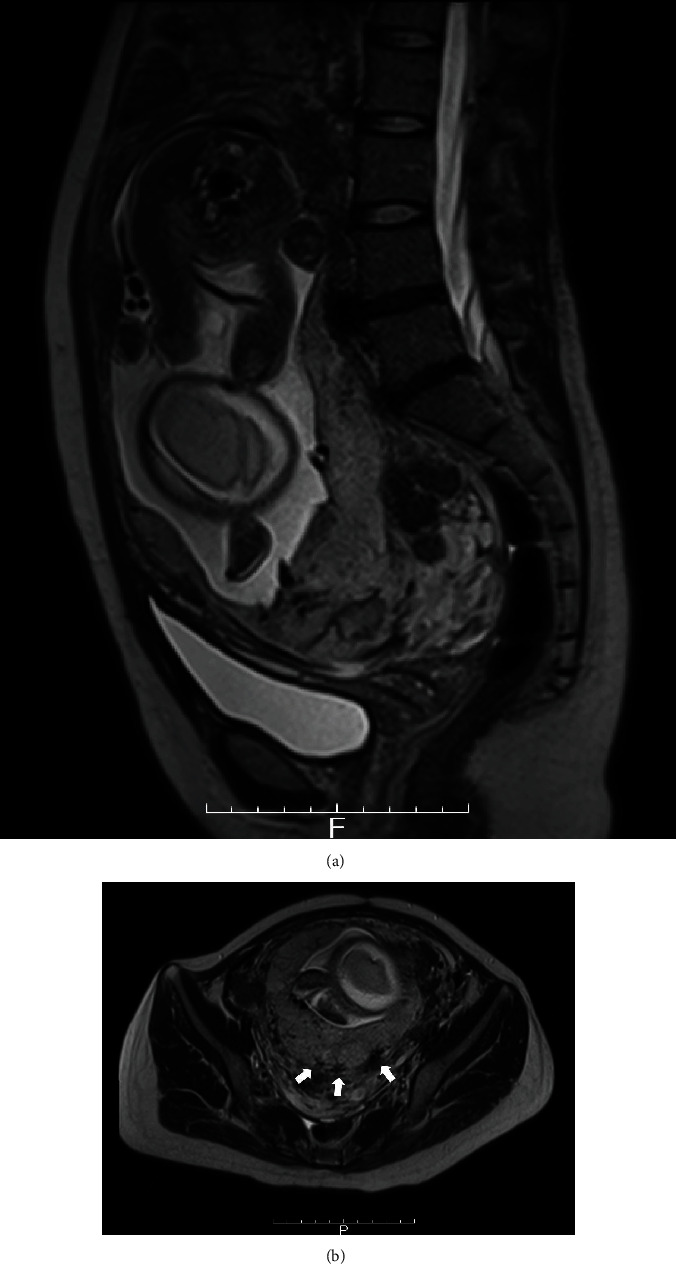
MRI findings of placenta previa. (a) Sagittal and (b) axial image. Arrows represent placental recess (a wedge-shaped contraction of the placental surface and uterine outer rim accompanying a T2 dark band).

Ultrasonography revealed a clear retroplacental hypoechoic zone, which did not suggest the presence of placenta accreta spectrum (PAS), whereas magnetic resonance imaging showed placental recess (a wedge-shaped contraction of the placental surface and uterine outer rim accompanying a T2 dark band), which is a predictive sign of PAS (Figure 3) [12]. Also, the placenta was present on the scar of the previous extensive myomectomy. If PAS is present, hysterectomy must be performed urgently in some cases [13]. However, hysterectomy is difficult to perform when the small intestine is attached to the uterus. Therefore, we scheduled her cesarean section for 32^+4^-week gestation because (i) fasting and TPN were too long and strong patient psychological stress and (ii) fear of massive bleeding due to placenta previa, which frequently occurs around 34 to 36-week gestation. An intra-aortic balloon occlusion catheter was inserted before a cesarean section to avoid massive hemorrhage in case of PAS. A cesarean section under general anesthesia was performed, and she gave birth to a healthy male weighing 1807 g (46.88 percentile) with an Apgar score of 4 at 1 min and 6 at 5 min. The placenta was spontaneously delivered without manual removal, and uterine hemorrhage was well controlled by prophylactic intrauterine balloon tamponade without inflating the intra-aortic balloon occlusion catheter. The total blood loss was 3100 g, and the patient received a blood transfusion. During laparotomy, there were ileal loops strongly adhered to the uterine scar of the myomectomy, but there was no volvulus of the small intestine. The patient underwent adhesiolysis and enterectomy. The patient recovered uneventfully, without recurrent bowel obstruction.

## 3. Discussion

Diffuse uterine leiomyomatosis is a rare condition in which innumerable, poorly defined, and small leiomyomas replace most parts of the myometrium, leading to uterine enlargement of uterus [1, 14]. This rare condition affects women of reproductive age, and is associated with menstrual pain, menorrhagia, and infertility [5]. Hysterectomy is the only definitive treatment because it is not easy to perform myomectomy on numerous nodules with indistinct margins [2]. In order to restore fertility in patients with diffuse uterine leiomyomatosis, many treatments have been tried as alternatives to hysterectomy. The uterine artery embolization (UAE) may be an effective alternative to hysterectomy and one of seven patients treated by UAE became pregnant 5 months after UAE treatment [15], although pregnancy after UAE treatment is controversial. High intensity focused ultrasound (HIFU) treatment has also been used as an alternative to hysterectomy [16]. Purohit et al. reported that HIFU treatment reduced 67.6% of uterine volume and improved normal menstruation, although they did not have pregnancy outcomes after HIFU treatment. Gonadotropin-releasing hormone analog (GnRHa) is widely used for diffuse uterine leiomyomatosis, and successful pregnancies after GnRHa administration has been reported, but it is primarily administered before myomectomy to control bleeding during myomectomy [5]. Hysteroscopic myomectomy has also been reported to recover fertility in patients with diffuse uterine leiomyomatosis [17, 18].

We have currently performed a new surgical treatment, extensive myomectomy to restore fertility in women with diffuse uterine leiomyomatosis. In this procedure, the uterus is deeply dissected longitudinally with complete opening of the uterine cavity after tourniquet for hemostasis [4, 6]. Since this surgical procedure has only recently begun to be reported, it is unclear to what extent it improves fertility. It has been reported that the pregnancy rate after myomectomy is lower the greater the number of myomas enucleated [19, 20]. In the future, it is necessary to compare the degree of fertility improvement between conventional myomectomy and this new procedure in patients with diffuse uterine leiomyomatosis. This method results in a more extensive uterine scar that may cause perinatal complications, including uterine rupture. Furthermore, myomectomy causes peritoneal adhesions that make cesarean section more difficult.

Small bowel obstruction during pregnancy is a very rare disease, with an incidence of 0.0014%–0.0034% [21]. A review of small bowel obstruction in pregnancy showed that 50% of all cases were caused by adhesions [8]. Surgical treatment was performed in 91% of adhesive small bowel obstructions because conservative treatment failed in 81% of cases. The problem lies in the high fetal mortality rate. The risk of fetal loss was 17% in patients with adhesive small bowel obstruction, whereas the rate of maternal mortality was 2% [8]. In the previous report, fetal deaths occurred when the maternal condition was severe and required emergency laparotomy in 2^nd^ and 3^rd^ trimester [22]. It suggests that the high fetal mortality rate is related to fetal-maternal infection rather than due to preterm delivery. In our case, the baby was born prematurely at 32-week gestation and grew without problems. However, unexplained oligohydramnios occurred for 10 days between 28- to 30-week gestations when the mother developed fever. A nonstress test and ultrasonography showed no abnormal fetal signs, except for oligohydramnios. Amniotic fluid gradually increased as the mother's condition improved with conservative treatment. Maternal dehydration may be the cause of oligohydramnios.

Myomectomy is considered a risk factor for PAS, which is a rare life-threatening condition of pregnancy [13]. PAS is the abnormal attachment of placental villi to the myometrium, which includes placenta accreta (attachment to the myometrium without decidua), placenta increta (invasion into the uterine myometrium), and placenta percreta (deeper invasion into surrounding organs, such as the bladder). Compared to conventional myomectomy, extensive myomectomy results in much longer uterine scar into endometrium, which may increase the risk of PAS. In our case, IVF pregnancy is also a risk factor of placenta anomalies [23]. The main clinical problem is massive bleeding, which occurs when the placenta does not detach after delivery. Hysterectomy is sometimes required when massive bleeding is not controlled by other hemostatic procedures. Placenta Accreta Spectrum Ultrasound Scoring System (PASUSS) can estimate the severity of PAS and can be a useful tool to predict massive bleeding during PAS [24].

Our case was a rare combination of placenta previa and adhesive small bowel obstruction, for which we were unable to perform immediate hysterectomy. We planned to perform a cesarean section with an intra-aortic balloon catheter to avoid emergent hysterectomy due to massive bleeding. Fortunately, the placenta detached normally, and intra-aortic balloon occlusion was not used. The total blood volume during the cesarean section and enterectomy was lower than expected. A lesson from our case was to perform immediate full preparation for the cesarean section. As previously described, small bowel obstruction during pregnancy is associated with a high risk of fetal loss [8]. When the maternal and fetal condition worsens, immediate laparotomy and fetal delivery are necessary to end the pregnancy.

This is the first case report of adhesive small bowel obstruction during pregnancy after extensive myomectomy for diffuse uterine leiomyomatosis, which is our strength of the case. However, the fertility-improving effects of extensive myomectomy are still unknown. Also, perinatal complications after extensive myomectomy will be reported. It is necessary to compare the degree of fertility improvement and perinatal complications between conventional myomectomy and this new procedure in patients with diffuse uterine leiomyomatosis.

We report a case of two rare complications, adhesive small bowel obstruction and placenta previa, presumably due to aggressive myomectomy for diffuse uterine leiomyomatosis. The placenta was not adherent to the scar of the previous extensive myomectomy in our case. However, the scar of extensive myomectomy is larger and deeper than that of conventional myomectomy, which may increase the risk of adhesive small bowel obstruction and PAS. Adhesive small bowel obstruction can lead to severe maternal conditions, sometimes resulting in fetal death. Pregnant women after extensive myomectomy should be managed with the possibility of adhesive small bowel obstruction in mind and the degree of placental adhesion to the scar should be evaluated.
